# From monochromatic waves to realistic tides: deep learning for short-term forecasting of coastal ocean

**DOI:** 10.1038/s41598-025-31670-2

**Published:** 2025-12-21

**Authors:** Irem Yildiz, Emil V. Stanev, Joanna Staneva

**Affiliations:** 1 Institute of Coastal Systems - Analysis and Modeling, Helmholtz-Zentrum Hereon, Geesthacht, Germany; 2 Department of Meteorology and Geophysics, University of Sofia “St. Kliment Ohridski”, Sofia, Bulgaria

**Keywords:** Deep learning, Tide, LSTM, CNN, Forecasting, Autoencoder, Climate sciences, Mathematics and computing, Ocean sciences

## Abstract

In this study, a hybrid architecture combining convolutional neural networks for spatial reconstruction and long short-term memory networks for temporal forecasting is used to predict sea-level variations in the German Bight. This new framework is applied to a series of sea level data ranging from academic to realistic data. Experiments with monochromatic waves demonstrate the model’s ability to deliver accurate short-term forecasts with minimal errors. Forecasts of real tidal constituents, including M2 and the sum of M2 and M4 tides, confirm robust model performance over lead times up to 48 h. A key result is that deep learning can reconstruct basin-wide sea level from a limited number of coastal gauge stations. Therefore, in the forecast experiments, adding data from coastal observations (mimicking data assimilation) significantly improves prediction accuracy. The study highlights the potential of deep learning to supplement traditional numerical models, particularly in regions with dense observational coverage. Key factors influencing model performance are identified, among them spatial signal complexity and steepness of gradients. An overall result is that deep learning can complement numerical models in operational ocean forecasting and provide a valuable tool for evidence-based coastal management in data-rich regions.

**Supplementary Information:**

The online version contains supplementary material available at 10.1038/s41598-025-31670-2.

## Introduction

Tidal forecasting has a rich history, beginning with the pioneering work of Lord Kelvin^1^, who applied Fourier analysis to understand tidal patterns. This foundational approach established the basis for modern tidal theory and forecasting. Later, Kelvin’s work was expanded by developing the tide-generating potential in a harmonic form^2^, providing more rigorous mathematical description of tidal forces. Building on these foundational concepts, significant strides were made in interpreting tidal observations, particularly in terms of the effects of wind and atmospheric pressure on sea-level fluctuations^3^. These early contributions laid the groundwork for modern tidal prediction approaches and deepened our understanding of the complex interactions between astronomical and meteorological forces.

In the mid-twentieth century, the focus shifted toward numerical modeling. The development of the first two-dimensional numerical storm surge models^4–7^ marked a significant leap forward in predicting coastal flooding. These models provided a framework for the development of early warning systems, essential for mitigating the risks of storm surges and other coastal hazards.

With the advent of satellite technology, complex numerical models, and the growing computational capabilities of modern computing systems, the accuracy of sea-level predictions has steadily improved. High-resolution models and data assimilation techniques enhance the ability of operational oceanography to provide timely and reliable forecasts of tides, storm surges, and other sea-level fluctuations^8,9^. These advances not only contribute to scientific knowledge but also can support practical applications in coastal management, disaster preparedness, and climate change adaptation.

One reasonable question in the era of artificial intelligence is whether data-driven methods can complement or enhance traditional deterministic modelling. In particular, it is relevant to ask whether deep learning (DL) approaches trained on observational or model-generated data can achieve forecasting skill comparable to physics-based numerical models used in coastal applications. This is an important consideration because tides, while well understood in principle, are influenced by complex local processes that challenge traditional models. Recent reviews^10–12^ demonstrated that machine learning (ML) methods applied to sea level can complement or even rival traditional approaches for specific coastal applications. Water level observations in single-station and convolutional neural networks (CNNs) were used to predict storm surges at several locations^13^. A long short-term memory (LSTM)-based forecasting model was used for tidal sea level prediction in 17 harbors in Taiwan^14^ and demonstrated superior performance in forecast accuracy compared to six other models. A bidirectional long-short term memory (Bi-LSTM) deep neural network model for predicting tidal water levels also demonstrated a strong short-term forecast skill^15^. In another application^16^, a combined model based on variational mode decomposition (VMD) and LSTM was proposed where VMD decomposed tidal signals into their components, which were then predicted individually with LSTM before recombination. Graph convolutional recurrent networks were used to predict tidal levels at regional multiple tide stations^17^. The model demonstrated superior performance in comparison to five commonly used baseline models. Very recently, a convolutional long short-term memory (ConvLSTM) model incorporating an attention mechanism for nearshore water level prediction was proposed^18^ that further improved model interpretability and forecast reliability. Almaliki and Khattak^19^ presented a hybrid Temporal Convolutional Network (TCN) and LSTM model capable of predicting both short-term and long-term tidal fluctuations. The study demonstrates the potential of a hybrid TCN + LSTM framework for reliable tidal level prediction, thereby supporting improved planning and decision-making in coastal and maritime applications.

Experience also exists in the frame of basin-wide tidal analyses using ML. For example, ML was used for detecting sea-level states with anomalous spatial correlations using autoassociative neural networks, trained with different sets of observation and model-based data^20^. The performance of the Kalman filter approach against generative adversarial networks was compared to reconstructing the sea-level variability in the North Sea using observations from 19 coastal tide gauges and data from numerical models^21^. The results showed that, for largely linear tidal dynamics, both methods achieved comparable performance, demonstrating that ML can replicate established techniques for such systems. The above brief overview of various ML applications dealing with sea level reconstruction and forecasting would not be complete without emphasizing that the skill of models based on ML is competitive to the skill of other methods used in oceanography and coastal engineering. This forms the basis of the main objective of this study, which is to evaluate basin-wide tidal forecasting using DL. A specific objective of this study is to determine the capabilities and limitations of DL in the coastal ocean. The success of DL techniques in reconstructing and forecasting sea levels would reflect their ability to learn the dynamic relationship between the coast and the open sea; in other words, the dynamics of continuum coast-open sea. This is of the utmost importance given the growing interest in developing coastal zone science and addressing environmental issues in this part of the World Ocean.

**Fig. 1:**
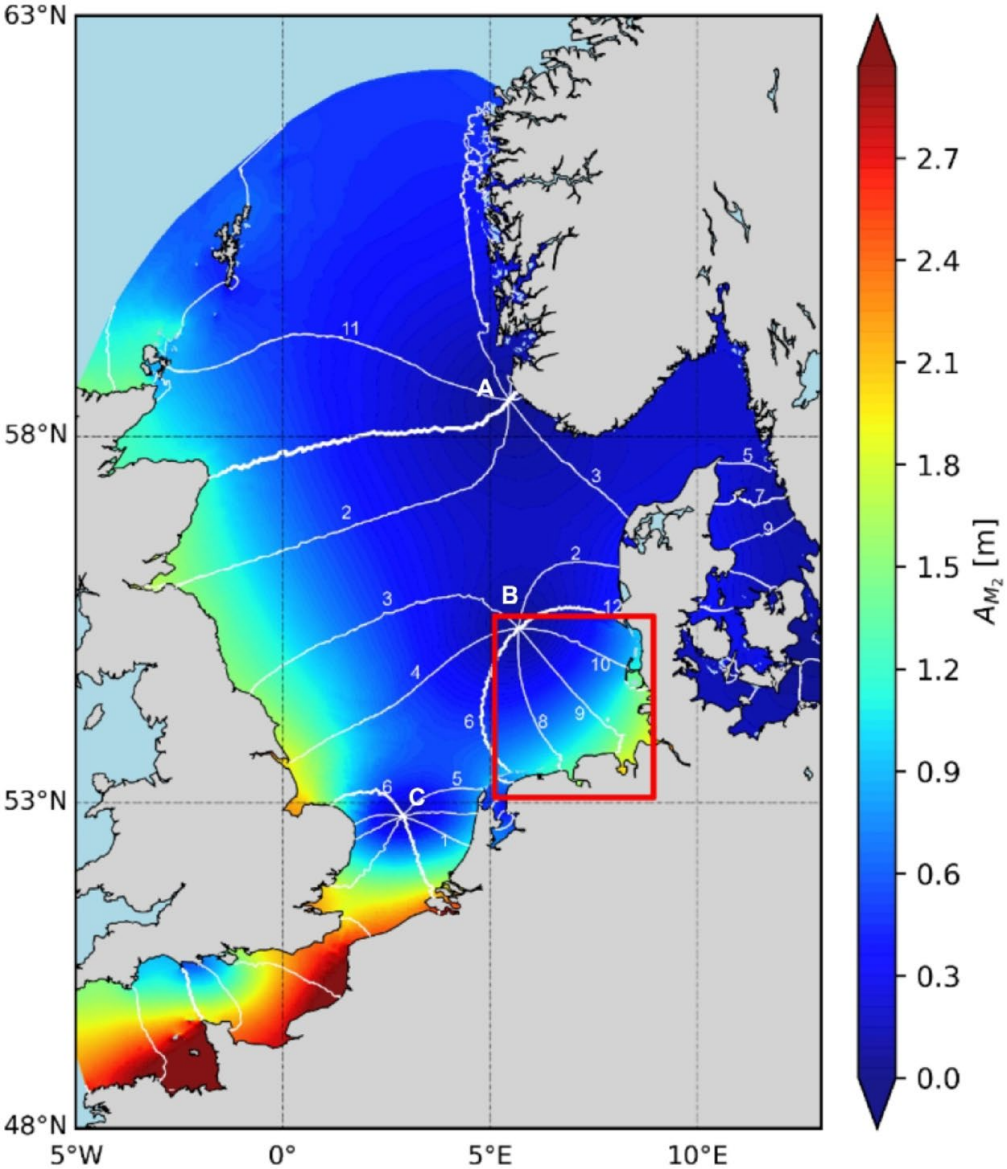
An overall presentation of tidal characteristics in the North Sea. The amplitude of the M2 tide (A_M2_) is shown with different colors. The white lines illustrate the phases—for example, each successive line indicates the time of high-water onset. The points A, B, and C, where these lines converge to a single point are the amphidromic points. The red box shows approximately the region studied here. Data used to plot this figure originates from the tidal analysis of sea level produced by the model of Jacob and Stanev^24^.

Here, we analyze the capability of ML to be used as a forecasting tool for the spatial and temporal variability of tides in the German Bight. This geographical area is part of the North Sea where tidal waves are consolidated around several amphidromic locations. In these locations, which are marked with the letters A, B, and C in Fig. 1, the tidal wave has a minimum amplitude. The wave rotates around these points, increasing in amplitude as it approaches the coast. The amphidromic point B is in the north-western German Bight. By combining CNNs for spatial reconstruction with LSTM networks for temporal forecasting, we develop an ML-based framework to predict sea-level variations in the studied area. The training data we use originates from numerical forecasts. As these data are used as artificial observations, the present study can be categorized as data-driven forecasting. The task set up here is to use past observations and train a ML model to forecast future states. Before moving to realistic scenarios, we also conducted a series of controlled experiments to systematically assess model performance across increasing levels of signal complexity. These cases vary from simple monochromatic waves to data derived from tidal analyses and real sea level data. This stepwise approach, from simple to complex tidal signals, provides insights into the limits of ML-based prediction and the influence of signal complexity on forecast skill. This exercise may appear didactic and not entirely consistent with the numerous applications of ML in geophysics, where scientists use this new technique to solve very complex problems^22,23^. However, it appears useful to (1) test the quality of performance of forecasting depending on the complexity of geophysical signals used, and (2) to explore the predictability in the range from simple to complex dynamics. We should also bear in mind that tides are not just simple waves; they are characterized by a wide spectrum of frequencies. This includes overtides caused by nonlinear processes. The coupling of tides with oceanographic processes of different origins, such as with currents or local dynamics affected by bathymetry and meteorological conditions adds another level of complexity. The approach to using data of different complexities, from monochromatic waves to realistic tides, also helps us understand the different limits of ML-based prediction in systems, which are rather well predictable using deterministic tools (e.g. numerical models).

Tide gauge stations operating along the North Sea coast provide locally high-quality records of sea levels over a long period^25^. However, it is still not clear whether using such data along with ML could contribute to improved tidal predictions basin-wide. This issue will be also addressed in the present study. We assess whether incorporating a limited number of coastal observations can enhance basin-wide forecasting skill, offering a practical approach for improving predictions in operational settings.

The paper is structured as follows. Section “Results” presents the results of the reconstruction and forecasting experiments across varying levels of signal complexity. Section “Discussion” provides an in-depth discussion of these findings in the context of forecasting and coastal applications. Section “Methods” describes the data, experimental design, and DL architecture used in this study. Finally, Section “Conclusions” summarizes the key conclusions and outlines directions for future research.

## Results

### Rationale

**Fig. 2:**
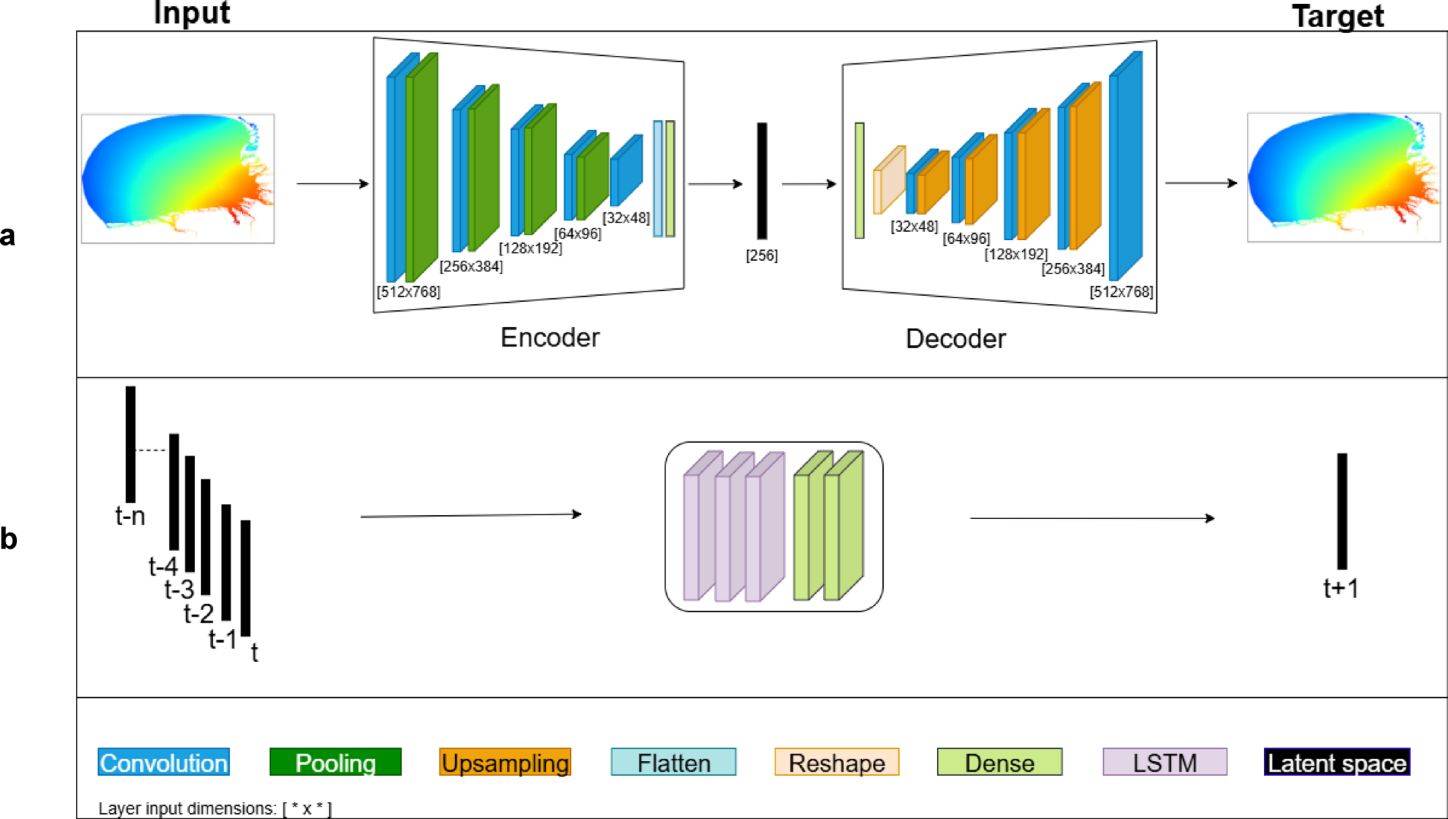
Implemented model architecture. (**a**) illustrates a convolutional Autoencoder architecture, which compresses the inputs into a latent space representation, (**b**) presents the LSTM-based architecture, receives the autoencoder’s latent features as input and forecast future step by modeling their temporal dependencies. The layers in (**a**) depicted in dark blue represent the convolution layers. The dark green color corresponds to the pooling layer, while dark orange one to the upsampling layer. The light green color represents the dense layer, while the light blue and the light orange ones are the flatten and reshape layers, respectively. The black bar between the encoder and decoder is the latent space. The lilac layer in Fig. 2b, is representative of the LSTM, while the light green layer corresponds to the dense layer.

This section evaluates the performance of the DL framework for sea-level reconstruction and forecasting in the German Bight. For our analysis we use sea level data produced by the Semi-implicit Cross-scale Hydroscience Integrated System Model (SCHISM), which was set up for the German Bight^26^, as well as simpler wave data generated analytically. The model-generated data, which is explained in detail in the Methods section, spans a period of 13 months and is recorded at hourly intervals. The DL models used were informed by the integration of temporal and spatial data. As illustrated in Fig. 2, a two-stage architecture was developed. The initial stage involves the implementation of a CNN-based model, while the next stage involves the implementation of an LSTM-based forecasting model. The initial stage performs a reconstruction of two-dimensional sea level, and the objective here is to obtain representations (latent space), which are a low-dimensional space representing compressed and meaningful features of the data, of the images. In the second stage, these latent spaces are leveraged for future forecasting. The root mean square error (RMSE) metric has been used to evaluate model performance. For more details, see the Methods section.

The experimental design, which is described below, first analyses idealized cases with simple waveforms in order to isolate fundamental model behavior. It then progressively introduces increasing spatial and temporal complexity, culminating in experiments that use realistic tidal variability. This sequence enables us to evaluate model performance under controlled conditions prior to applying it to real coastal observations.

### Experimental design

#### Reconstruction of 2D fields

**Fig. 3:**
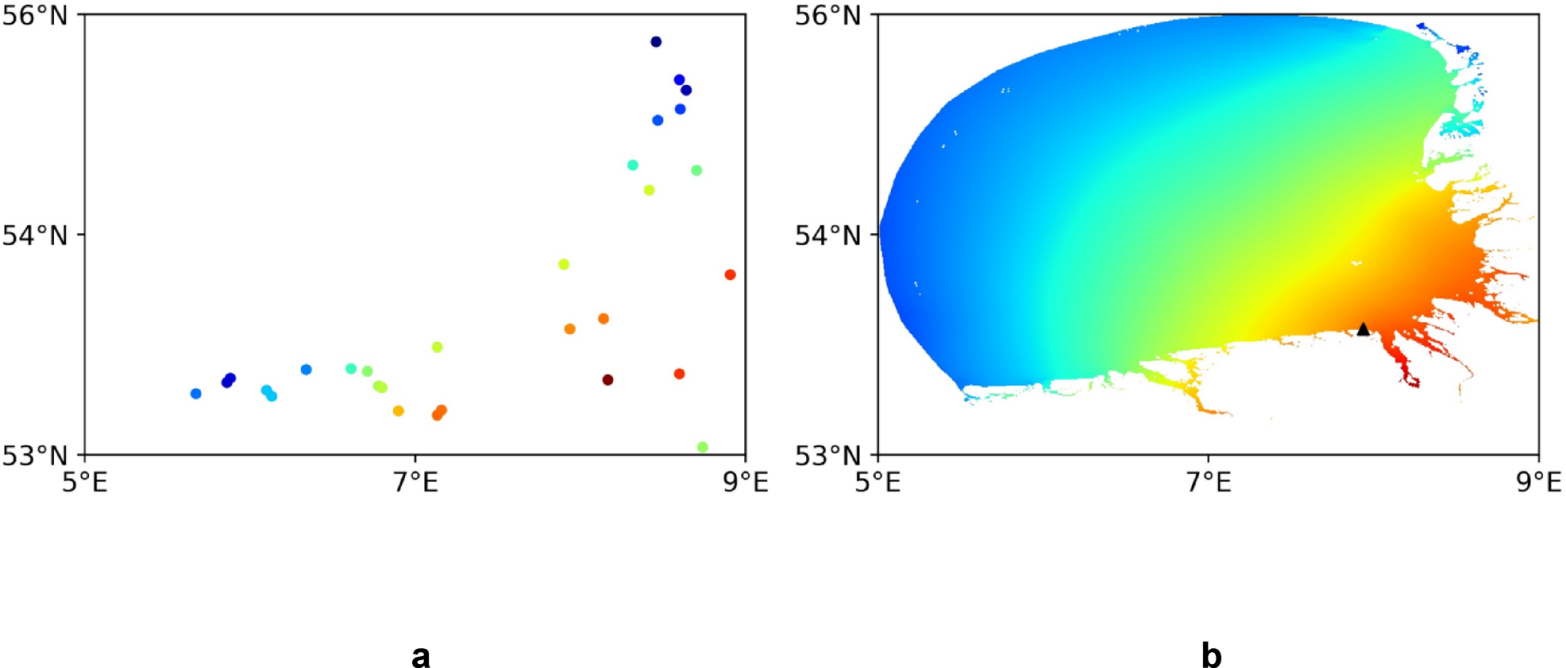
Input data (**a**), and target data (**b**) in the reconstruction experiment. Triangle symbol in (**b**) illustrates position of data used in one of the experiments (see Section “Improvement of tidal forecasts by using data in future times”).

The task here is to quantify the quality of the reconstruction of the 2D structure of the tides using ML. We are following the approach proposed in^21^, which aims to reconstruct sea-level variability in the North Sea using coastal observations and data from numerical models. The difference is that our current application is for the German Bight area, and that we use a different method (see Methods section). In the learning phase, we use data in 29 locations of tidal gauges (Fig. 3a) as input (forcing) data, and the full 2D data set as described in the Methods section as target data. The 2D reconstruction uses the autoencoder architecture shown in Fig. 2a. Input and target data for one snapshot are shown in Fig. 3.

#### Forecasting

##### Multi-step forecasting

Forecasting experiments include the architecture shown in Fig. 2 with data sets in all experiments covering a matrix of 512 × 768 pixels (the horizontal resolution is 330 $m$ in x-direction and 550 $m$ in y-direction) and with the same temporal resolution (hourly). Forecasts for time $t+1$ using information from *n* previous time steps, (see the Methods section for a detailed description of the forecasting procedure), make it possible to develop multi-step forecasting through the implementation of a recursive method. This method first predicts a value at time $t+1$ and then uses it as a known observation, along with the previous observations (from time $t-n+1$ to time *t*) into the forecasting model to obtain the forecast value at $t+2$. Through this iteration, the forecast value is obtained up to time $t+m$, where $m$ is the number of predicted future steps, thus achieving the multi-step forecasting^27^. The obtained multi-step forecasting latent spaces are converted into two-dimensional form by passing them through the decoder in the first stage.

In all forecasting experiments described below, 96 past observations (time steps), that is four days, have been used to forecast the future states of sea level recursively. We remember that in the recursive method, the first of the 96 data is forgotten, and the newly computed value at time* t* + 1 is taken as observation in the next cycle of forecasting. Because the forecasted sea level differs from the original data, it is expected that the quality of model performance would decrease with time progressing.

##### Experiments

Four different data sets were first generated over 13 months, corresponding to the output from the numerical model discussed in the Methods section, as shown in the first four lines in Table 1. The first 12 months were reserved for training and validation, the rest for testing. The same set of parameters (see Methods) was used in all experiments.

**Table 1:** Nomenclature of all experiments.

	Experiment description
FI1	Monochromatic wave
FI2	Superposition of two linear waves
FI3	Wave with spatially variable amplitude
FI1-L	Monochromatic wave (longer wavelength)
FIT	Sea level as a rotating plane
FM2	M2 tide
FM2M4	M2 + M4 tides
FRT	Realistic sea level
FRT-A	Coastal data added to FRT
FRT-G	Only data from tidal gauges are used

To improve interpretability, individual experiments are categorized by increasing signal complexity, starting with idealized waveforms and progressing toward realistic sea level (Table 1). We first carried out three idealized experiments, in which (1) the waves were specified as monochromatic,

1 $$\eta = A\cos \left( {k_{x} x + k_{y} y - {\upomega }t} \right)$$

with A = 1 m, wavelengths in x and y direction equal to the zonal and meridional extent of the model area, and a period of 12 h, (2) a superposition of two linear waves (analogy to M2 plus M4 tide) as

2 $$\eta = A\cos \left( {{\text{k}}_{{\text{x}}} {\text{x}} + {\text{k}}_{{\text{y}}} {\text{y}} - {\omega t}} \right) + \frac{{\text{A}}}{4}\cos \left( {2{\text{k}}_{{\text{x}}} {\text{x}} + 2{\text{k}}_{{\text{y}}} {\text{y}} - 2{\omega t}} \right),$$

and (3) a monochromatic wave as in (1), but with spatially variable amplitude as

3 $$A\left( {x,y} \right) = \frac{1}{2}\left( {\frac{{X - X_{0} }}{{X_{{{\text{end}}}} - X_{0} }}} \right) + \left( {\frac{{Y - Y_{0} }}{{Y_{{{\text{end}}}} - Y_{0} }}} \right).$$

We call for brevity these idealistic experiments FI1, FI2, and FI3 (“F” and “I” stand for forecasting and idealistic), respectively. We repeated experiment FI1 specifying the wavelength as five times longer than in FI1 experiment and call it FI1-L (“L” stays for long).

Before switching to using realistic tidal signals, we did an experiment with a simplified tide specified as a plane rotating around an amphidromic point.

4 $$\eta = \left( {\frac{y}{\left| y \right|}} \right)\left( \frac{y}{b} \right)^{2} \cos \left( {\omega t} \right) + \left( {\frac{x}{\left| x \right|}} \right)\left( \frac{x}{a} \right)^{2} \sin \left( {\omega t} \right)$$

where *a* and *b* are half-length of the field along the axes. Here the size of the area where we compute $\eta$ is double the size of the remaining I-experiments. Therefore, we take only data to the south and east of the amphidromic point, thus the computational domain is identical with the one of the remaining I-experiments and the wave propagates in a similar way as in the studied realistic area (the amphidromic point is in the north-west corner, see also Fig. 1). In the nomenclature of experiments, this experiment is named FIT experiment, where “T” stays for tide. Movies of data used in the five I-experiments are shown in the supplementary material SI-1.Mov1- SI-1.Mov5 (the left most movies).

As a next step, the model output of sea surface height described in Methods Section (Data) was subjected to tidal analysis using UTide of python package. In the next experiment called FM2 we developed a model which forecasts the M2 tide. Experiment FM2M4 forecasts the sum of M2 and M4 tides. In the next experiment called FRT (forecasting realistic tides), we use the full tidal signal (realistic sea level).

In the next experiment FRT-A, we use coastal data in the future to “improve” the model performance. This approach, which resembles data assimilation, is aimed to “compensate” for the deterioration of the quality of forecast with prediction time increasing by adding observations into the forecasting procedure. Finally, in experiment FRT-G, the task is to make an hourly prediction of tides using only data from tidal gauges as observations from the past (“G” stands for gauges). We recall that in the reconstruction experiment tides were reconstructed for the time when gauge observations were taken; FRT-G makes one step ahead prediction. The nomenclature of all experiments is given in Table 1; the respective movies can be found in the supplementary material.

### Performance of deep learning applications

#### Reconstruction of basin-wide tides using data from tidal gauges

**Fig. 4:**
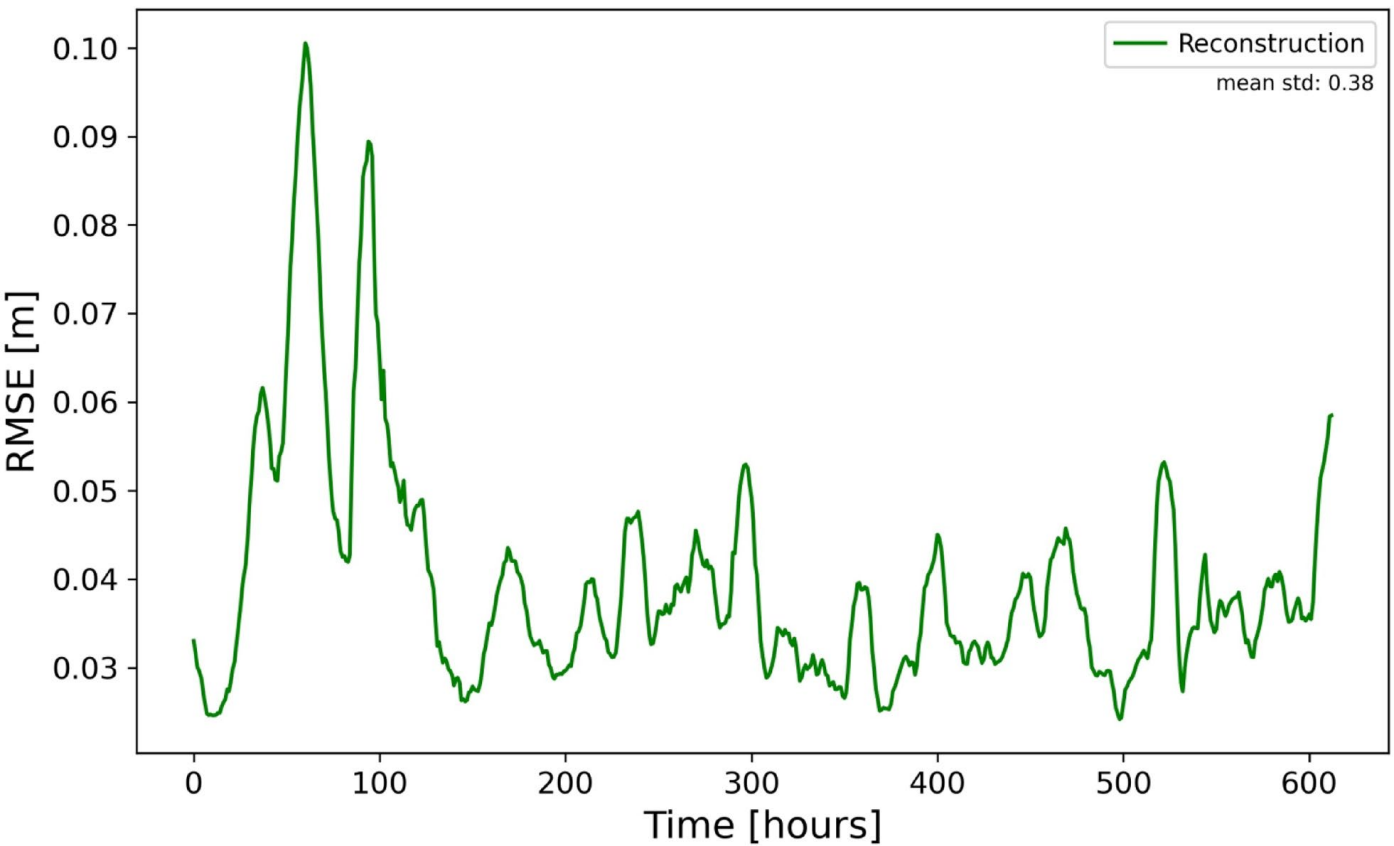
The quality of reconstruction, expressed as the area-mean RMSE (reconstruction versus test data). Mean standard deviation (std) of the test data is shown in the upper-right corner. The X-axis represents time in hours. Hourly data is plotted here and in all subsequent similar figures.

In the following, we present the quality of reconstruction of 2D sea level using coastal data only (see Fig. 3 for the design of this experiment). The DL model demonstrated strong reconstruction capabilities, effectively learning the spatial relationship between coastal gauge data and the full basin field. The quantification of the quality of reconstruction is done using independent data for one month (Fig. 4). The averaged RMSE for the test period is ~ 4 $\text{cm}$, which is small compared to the amplitude of the signal in the entire area (~ an order of magnitude larger). Some peaks of the error (~ 10 $cm$) occur for specific atmospheric situations.

#### Forecasting tides

In this section, we evaluate the predictive skill of DL models for progressively increasing the complexity of signals, from idealized waves to realistic tidal dynamics. This allows us to assess the scalability and robustness of the model architecture for various forecasting contexts.

##### Forecasting idealistic linear waves

**Fig. 5:**
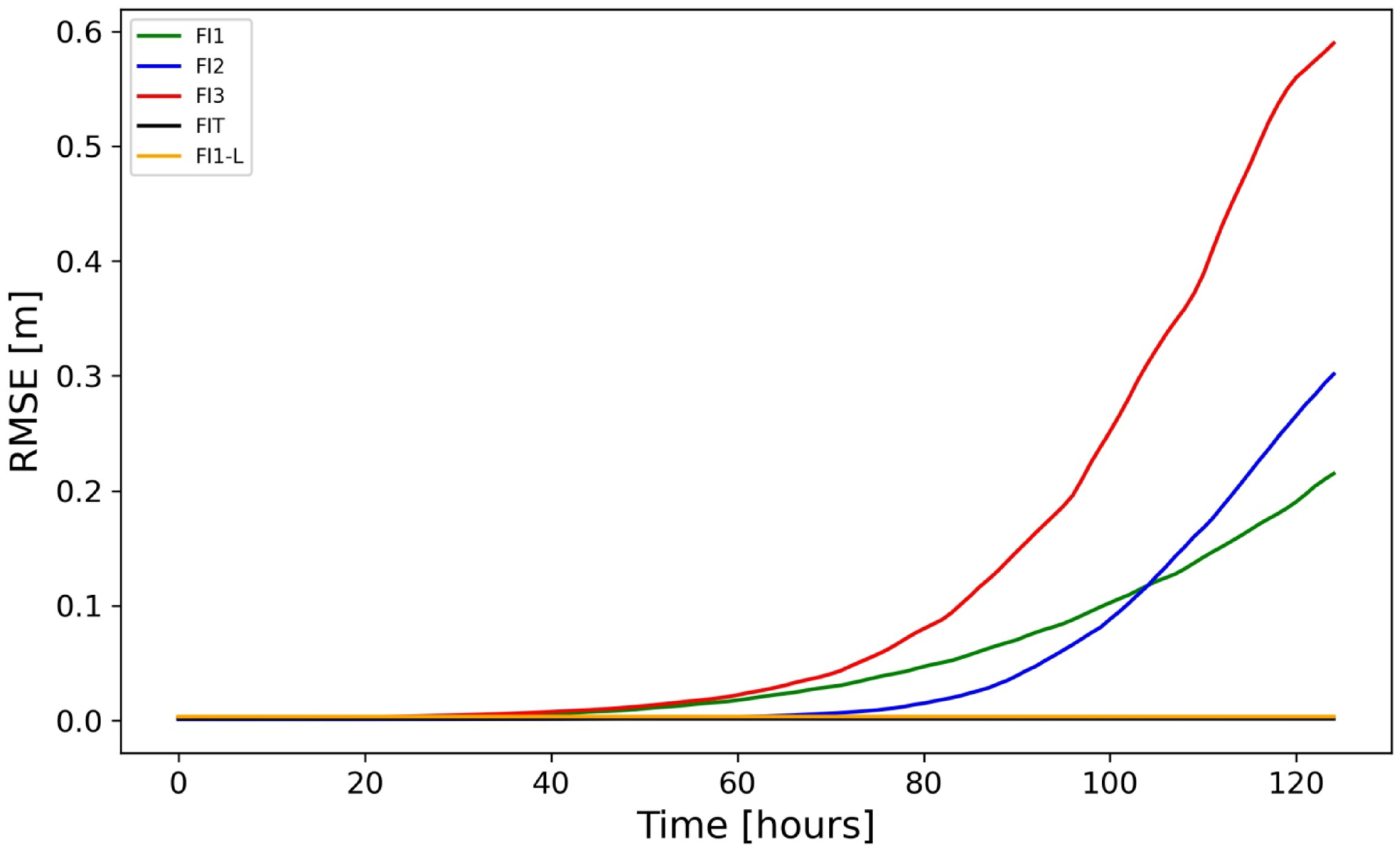
The predictive performance for experiments FI1, FI2, FI3, FI1-L, and FIT presented as the RMS difference between forecast and test-data. The forecast represents a multiple forecast obtained through recursive forecasting.

The RMSE when comparing the observations (test data) and forecast at time t + 1 in experiments FI1, FI2 and FI3 are 0.0028, 0.0011, 0.0016 $\text{m}$, respectively. Up to 125 h of multi-step forecasting was performed for these experiments. The respective RMSE values are shown in Fig. 5. As expected, forecast error accumulates slowly, and beyond 3 days-time, it increases exponentially in all three experiments. This increase is particularly strong in FI3 experiment demonstrating that the spatial inhomogeneity of signal reduces the quality of predictions. Notable is the different pattern of increase in errors in three experiments in the “exponential phase” when error approaches the amplitude of the real signal. Up to two days, the prediction is almost perfect in all three experiments.

In experiment FI1-L, the prediction is perfect at all times (see the Discussion section for the explanation). This suggests that for slowly varying or low-gradient signals, DL maintains forecast stability over extended lead times. Movies of original data in the I-experiments, predictions and respective differences can be found in the supplementary material (SI-1.Mov1- SI-1.Mov4).

The FIT experiment also belongs to the class of idealistic experiments. In this experiment prediction is almost perfect over a long period (Fig. 5 and SI-1.Mov5). This experiment bridges the gap between purely monochromatic waveforms and realistic tidal fields, offering insights into DL performance for simplified tidal dynamics.

##### Forecasting M2 tide and combination of tidal constituents

**Fig. 6:**
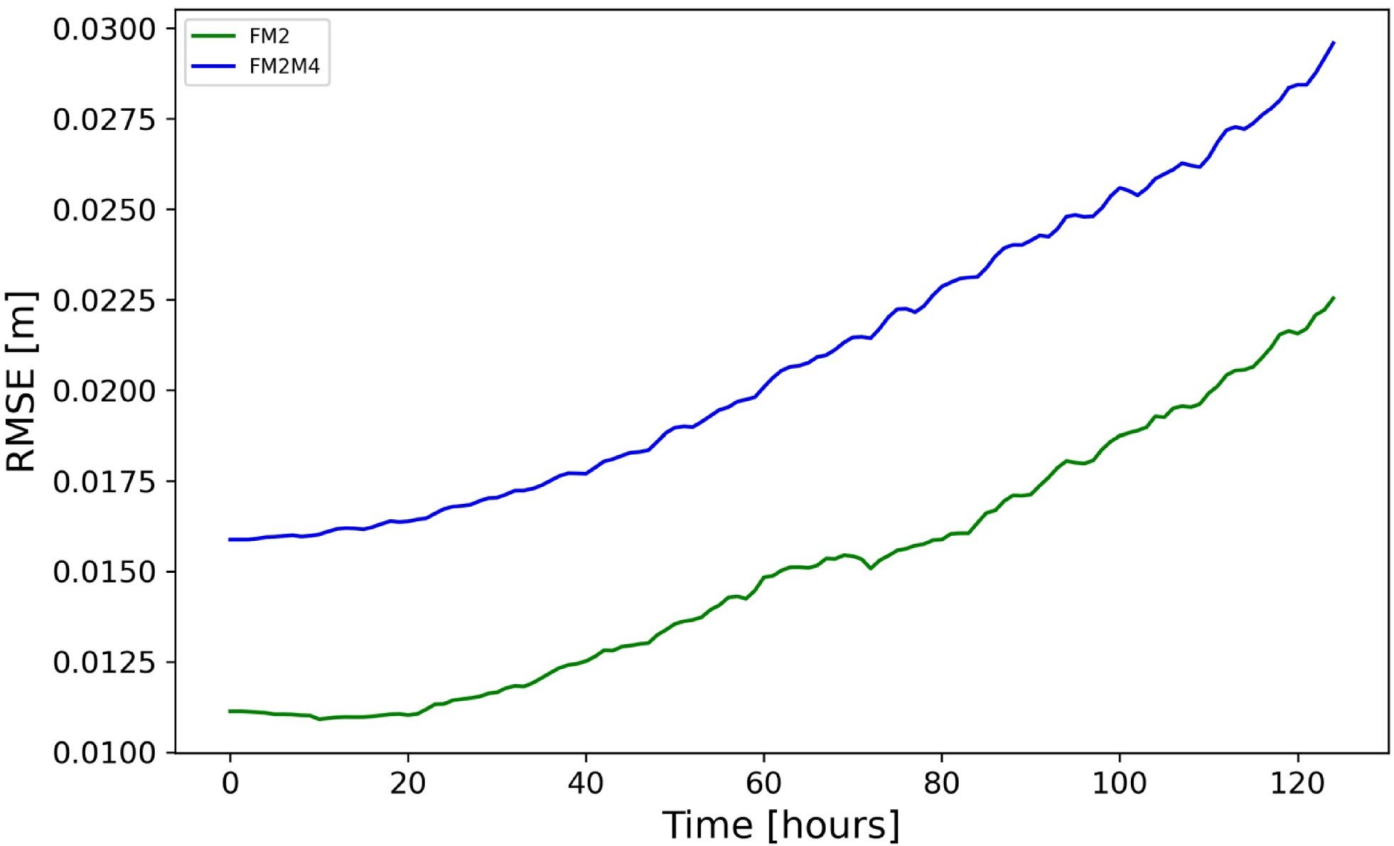
Predictive performance, as in Fig. 5 (RMSE of forecast versus test-data), in the experiments FM2 and FM2M4.

The RMSE of the forecast measured against the test data at time t + 1 is 0.011 $m$ for experiment FM2 and 0.016 $m$ for experiment FM2M4. Like in the case of FI1, FI2, and FI3 experiments, recursive multi-step forecasting was also conducted. The hourly RMSE values of FM2 and FM2M4 experiments for 125 h ahead are displayed in Fig. 6. Forecasting skill remains high for up to 48 h, with slower error growth. The more complex signal, two tidal frequencies and the insufficient temporal resolution of M4 tide (hourly), resulted in slightly reduced forecasting performance in FM2M4 experiment in comparison to FM2 experiment. Nevertheless, the error is more than an order of magnitude smaller than the amplitude of M2 tide. These findings indicate that the DL model can handle multi-constituent tidal dynamics. Movies of data, forecasts and errors in these experiments can be found in supplementary material (SI-2.Mov1, SI-2.Mov2).

##### Forecasting realistic sea level

**Fig. 7:**
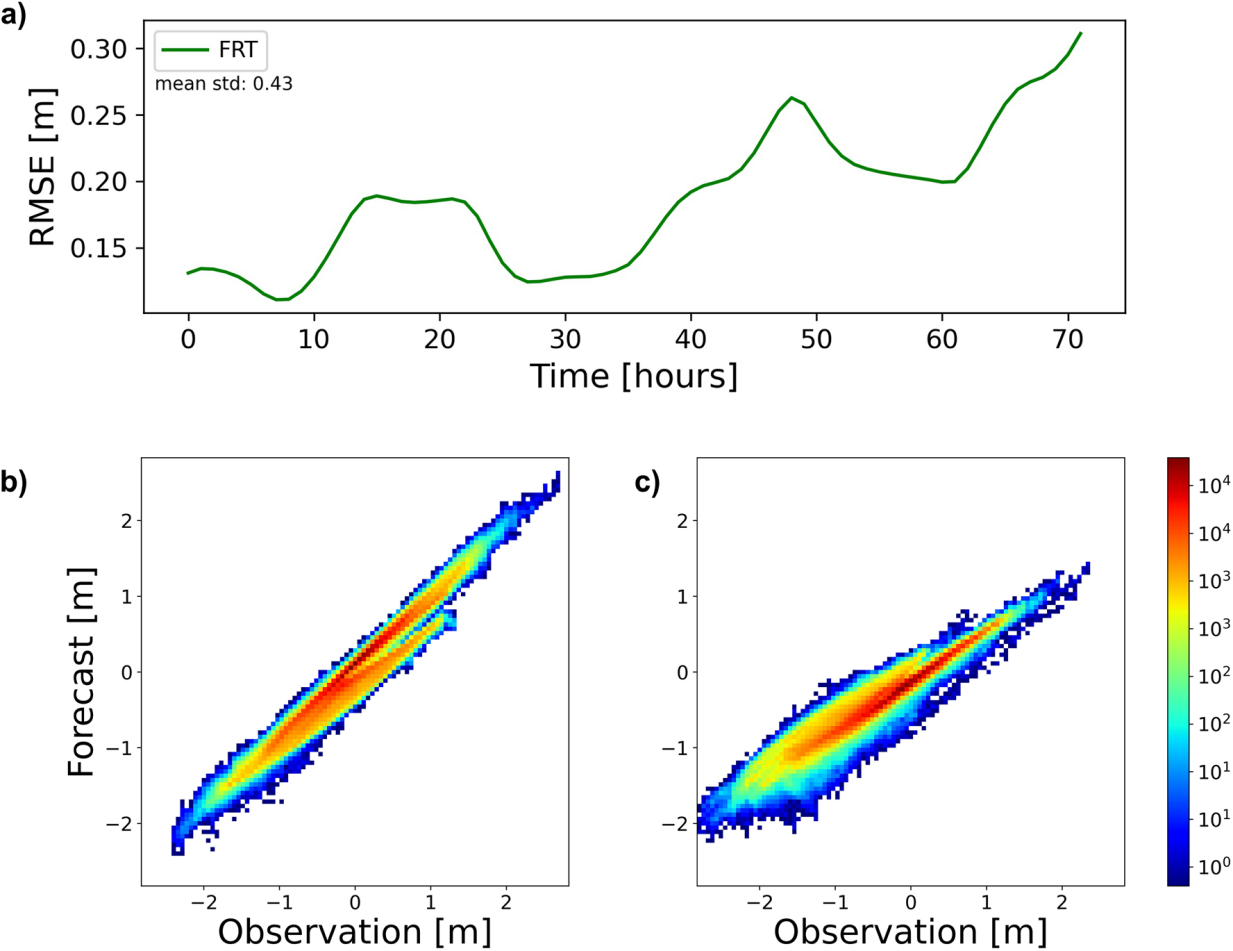
(**a**) Predictive performance as in Fig. 5 in the FRT experiment. (**b**) and (**c**) are pixel-based comparison of observation versus predictions from hour 1 to 12 (**a**) and from hour 36 to 48 (**b**). Color bar shows the number of matchups in individual bins (bin size is 0.06 $m$). Mean std measures the hourly standard deviation of the data.

In the FRT experiment, the full sea level data set was used for model training. The RMSE for time step t + 1 (one hour prediction) is 0.035 $m$. Over the next 72 h, recursive multi-step forecasting was conducted. The hourly RMSE values are displayed in Fig. 7a as a function of prediction time. A movie of original data, predictions and respective differences can be found in the supplementary material (SI-3.Mov1). An overall idea of the quality of forecast is given in Fig. 7b, c. The scatter plots display predicted and observation matchups in the first 12 h and from hour 36 to 48. The conclusion from this experiment is that the developed predictive model works satisfactorily for prediction times up to about two days.

##### Improvement of tidal forecasts by using data in future times

Led by the results illustrated in Fig. 4 (very low error in the reconstruction of the 2D field when using data only from the coastal stations), we carried out an additional experiment (FRT-A), in which data from one costal location was added as a predictor for future times. We used the location represented by a triangle symbol in Fig. 3b. To integrate the data at this location as a secondary input, it was inserted into the architecture depicted in Fig. 2b via a dense layer. Subsequently, the LSTM output of the architecture was concatenated with the output of the dense layer.

**Fig. 8:**
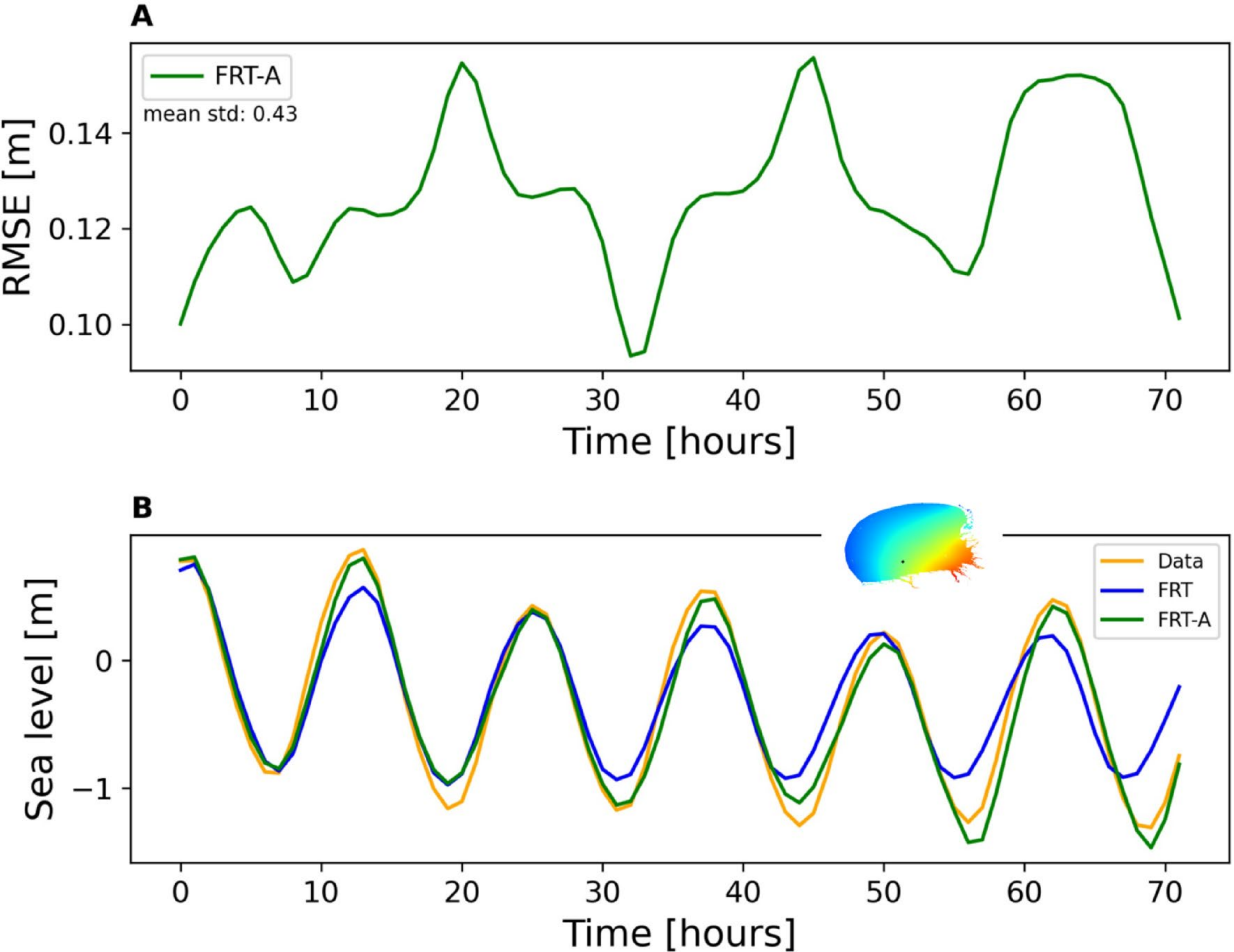
(**a**) Predictive performance as in Fig. 5 (RMSE) in the FRT-A experiment. (**b**) shows observations and predictions in the FRT and FRT-A experiments in the location shown as black point on the small map.

We hypothesize that the sea level at the relevant point was already known at the specified moment in time, and the t + 1 information from the coastal station was employed as an input to train the model. The RMSE value of the test data for moment t + 1 is 0.034 $m$ (0.035 cm in FRT experiment). However, up to 72 h later, the hourly RMSE values of the multi-step forecasting shown in Fig. 8a are substantially lower than those in the FRT experiment (compare with Fig. 7a). The temporal and spatial performance of the model is shown in the supplementary material (SI-4.Mov1). Figure 8b shows as an example the sea level at one location (the black point on the small map), from observations and model outputs of the experiments FRT and FRT-A. A comparison of the RMSE of FRT and FRT-A experiments over a longer period can be found in the supplementary material (SI-4. Figure 1).

##### Basin-wide tidal forecasts using coastal data only

**Fig. 9:**
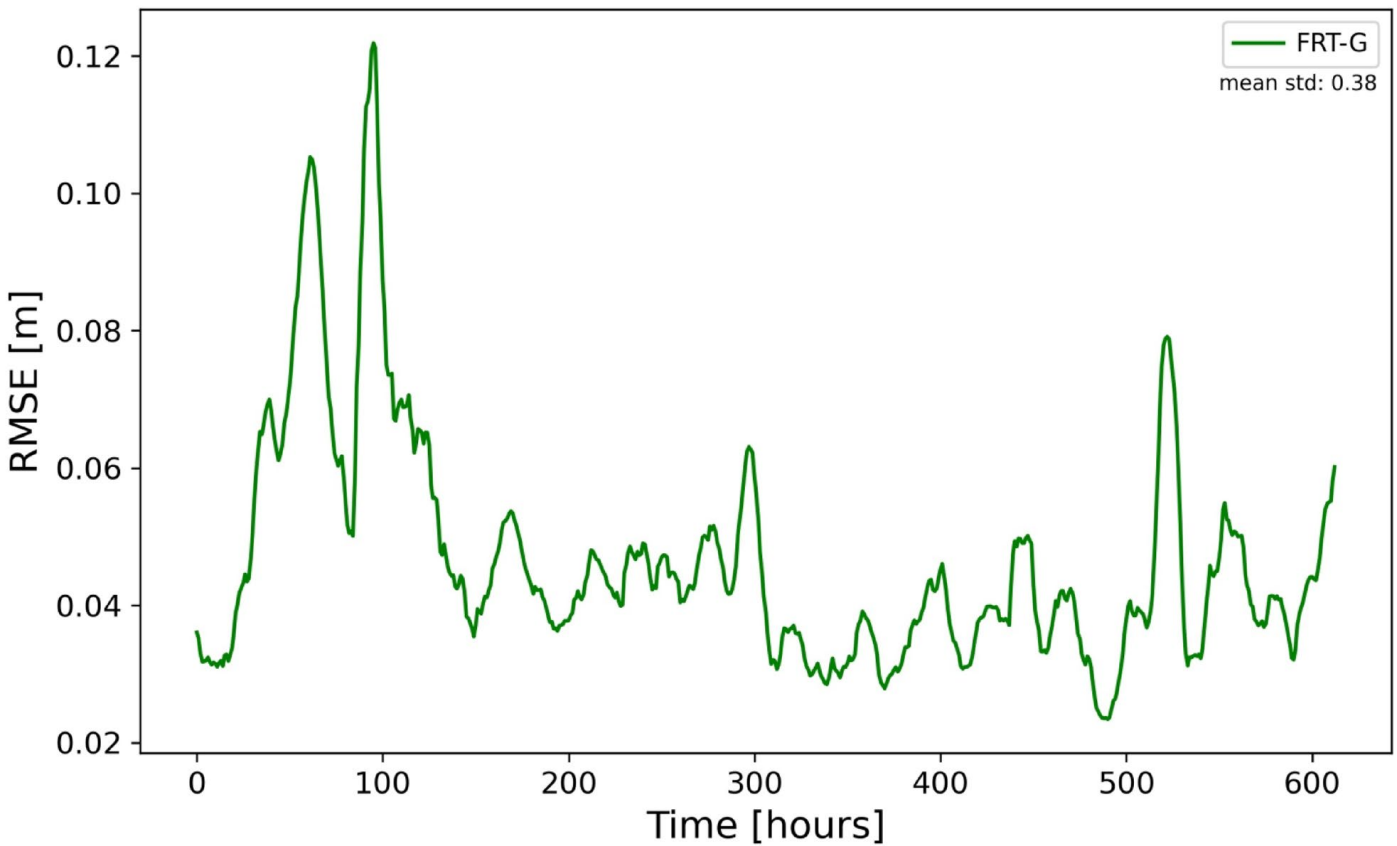
Predictive performance in experiment FRT-G. The basin-mean RMSE in the case of forecast using coastal data.

Led by the good performance of DL in the reconstruction of basin-wide sea level using data from coastal gauges only, as well as by the good performance of experiment FRT-A, we ask here whether we can use coastal data only for producing adequate basin-wide tidal predictions. In the next experiment FRT-G, we use the sea-level data from 29 coastal stations (See Fig. 3) to forecast the basin-wide sea level. The result of t + 1 forecasts using 96 time-steps in the past is shown in Fig. 9 and is compared with the reconstruction (Fig. 4). Obviously, the skill of the forecast is slightly lower than that of the reconstruction, nevertheless it is quite good. This illustrates the possible usability of real coastal observations for data- driven forecasts of sea level in the German Bight.

#### Comparative summary and operational implications

Across all experiments, the DL framework demonstrated strong performance in reconstructing and forecasting sea-level variability, with skill decreasing as signal complexity increased. Idealized experiments (FI1–FI3, FIT) showed that the model effectively captures simple wave dynamics with minimal error growth over multi-day forecasts. Forecasting of individual and combined tidal constituents (FM2, FM2M4) revealed robust predictive skill for up to 48 h, though the addition of higher harmonics slightly reduced accuracy. In fully realistic scenarios (FRT), forecast errors increased more rapidly, but incorporating future coastal observations (FRT-A) significantly mitigated this degradation, highlighting the value of observation-enhanced approaches.

Operationally, these results suggest that DL models can supplement or partially replace physics-based models for short-term tidal forecasting in coastal regions, especially where dense tide gauge networks exist. The ability to reconstruct basin-wide fields from limited observations further enhances their utility in data-sparse environments, providing actionable insights for coastal management and early warning systems.

## Discussion

### Applicability of deep learning for coastal forecasting

We want to know whether it is possible to learn the behavior of a dynamical system without knowing its underlying evolution equations. More generally, the same question posed for the atmosphere^28^ is: “Can models that are based on DL and trained on atmospheric data compete with weather and climate models that are based on physical principles and the basic equations of motion?”. The cited study examined global weather forecasting on the basis of experiments with geopotential height at 500 hPa. Similarly, in our study, we explored whether DL can be used to forecast the evolution of sea level in coastal systems without relying on physical governing equations. By focusing on a range of test cases—from idealized waves to full tidal signals—we assessed how DL models respond to varying levels of signal complexity. This strategy also reflects current trends in geophysical ML, where simpler datasets are used to isolate the intrinsic learning capabilities of the models.

In this article we investigate the applicability of DL to be used for data-driven forecasts using signals of different complexity and characteristics. Geophysical data involves multiple scales in length, time, and energy, as well as natural or instrumental noise. This issue is not entirely new; Faranda et al.^29^ address different strategies to overcome limitations of recurrent neural networks, when dealing with short-term forecasts as well as for reconstruction. In a future study, we will apply the developed methodology to sea level oscillations caused by storm surges and seiches in tidal and non-tidal basins, and the methodology will be extended to incorporate wind and atmospheric pressure as additional forcing.

In data-driven forecasting, training data comes from observations and is considered true. Sea-level observations are available from tidal gauges, but these only cover a few locations. Basin-wide data are available from altimeters, but the repeat time is too long and the quality of these data in coastal regions is poor. Ideally, we would have high-quality altimeter data from a satellite “hanging” over the German Bight measuring the sea level every five minutes. As such data is unavailable, we used model data as the truth (i.e. perfect data) and analyzed the predictive capability of the DL model. However, the question of how the predictive capabilities of DL compare against other tidal predictions across the basin still remains. We remember that our ‘perfect data’ are produced using Copernicus products for the North-Western European shelf as sea-level forcing at the open boundary. The relatively coarse resolution of the Copernicus model is inadequate for resolving all the important processes in the German Bight, a task that the SCHISM model performs well. The basin-mean RMS difference between the two simulations (Copernicus and SCHISM) is ~ 10 cm, increasing to more than 30 cm in the south-east corner of the German Bight. While these figures do not provide direct insight into the comparison between data-driven DL predictions and those based on numerical modelling, they do highlight differences between the models. These differences exceed the errors in our DL predictions, as demonstrated in the present study.

### Forecast skill and role of signal steepness

It is well-known that the skill of DL-based predictions depends on the parameters that are optimized for specific data sets. In our case we decided to use fixed combinations of model parameters for all data sets. Otherwise, one could generate an infinitely large set of experiments. The strategy of using the same parameters in all experiments, which were determined for the case of the FRT experiment, enables us to draw some conclusions about the capability for short-term predictability of different physical states when fed by long-term features of the respective data sources.

What appears interesting is that the increase of prediction error with increasing prediction time follows very different patterns in different experiments. In three I-experiments, the growth of error is exponential, which is similar to the case of dynamical systems. A four-day forecast in these experiments shows errors of ~ 5 cm, while in the case of the FM2 and FM2M4 experiments the error is about half of this value and its increase in time is not so steep as in the I-Experiments. This may be due to the DL system being optimized for the FRT experiment, which is similar to FM2 and FM2M4. However, our experiments suggest a more important reason: in FI1-L, where the wavelength is five times longer, the error is negligible. Obviously, less steep waves are forecasted almost perfectly.

### Comparison between idealized and tidal experiments

The FIT experiment is the most similar I-experiment to the tidal experiments. The similarity between FM2 and FIT experiments is that, in both experiments, the sea surface rotates around the amphidromic point. The major difference is that the ocean surface in the FIT experiment is described as a rotating parabolic surface, while in the FM2, the slope of sea-surface increases with approaching the coast as in a Kelvin wave. As seen in Fig. 5 the prediction in the FIT experiment is also perfect. The FIT experiment can be considered as a “transition” from I-experiments to experiments with realistic tides. However, there is another “transition” that is from the FI1-L experiment to tidal experiments and this is that in the FI1-L experiment the steepness of sea level (low) is comparable with the ones of real tides. This explains the perfect predictions in the FI1-L experiment.

## Methods

### Data

**Fig. 10:**
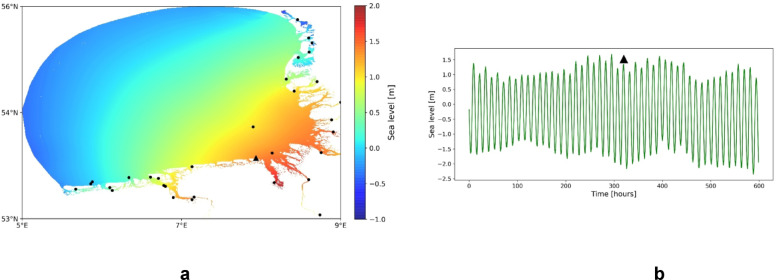
(**a**) Snapshot of sea level in the model area of the study^26^. Dots show positions of available tidal gauges. (**b**) shows exemplary the temporal change of sea level in the location shown in (**a**) by the big triangle symbol. The time in (**a**) corresponds to the position of the triangle symbol in (**b**).

SCHISM, which is an unstructured-grid model^30^, was used by Stanev et al.^26^ to simulate dynamics in the German Bight and its estuaries (Fig. 10a). The model solves the Reynolds-averaged Navier–Stokes equations, which are used in in hydrostatic form with the Boussinesq approximation. Implicit time stepping enables efficiency and robustness. The Eulerian–Lagrangian Method (ELM) is used for momentum advection, and the equations of salinity and heat transport are solved with a 2nd-order TVD method. Altogether, there are ∼475 K nodes and ∼940 K triangles in the model area. Estuaries are resolved with quadrangular grid elements, the narrowest of which are ~ 50 $m$ wide. The coarsest horizontal resolution is ~ 400 $m$ in the open sea. The vertical grid uses terrain-following S coordinates. The maximum depth is ~ 40 $m$.

The model uses realistic forcing data: hourly wind, atmospheric pressure, air temperature, dew point temperature and cloud cover. Forcing data originate from the 7 km-resolution COSMO-EU model of the German Weather Service (DWD). Freshwater fluxes from the estuaries of rivers Ems, Weser and Elbe are based on daily observations. At the open boundary, hourly values of elevation, horizontal velocity, salinity, and temperature from the Copernicus product (http://marine.copernicus.eu/) are used. A detailed analysis of model performance is presented in^26^. The numerically simulated data, which we analyze are for the period from 02.01.2017 to 31.01.2018. They are re-mapped in a uniform horizontal grid with a grid-size of 330 $m$ in x-direction and 550 $m$ in y-direction. Due to the very specific characteristics of Wadden Sea, which falls dry during part of the tidal cycle, we exclude from the analysis land pixels, or locations where in more than 11 percent of the time the bottom is dry (see Fig. 10a for the data coverage). Figure 10b shows part of the data record in one location.

The oscillations of sea level in the German Bight are sampled in a dense network of tidal gauge stations (Fig. 10a). These have been used by Stanev et al.^26^ for the validation of the numerical model presented shortly above. We will be using this observational network (positions in which we assign model data) for the reconstruction of sea level in the open sea, as well in support of tidal forecasting.

### Deep learning methods

#### Spatial reconstruction of 2D fields

DL methods are employed in the present study to forecast both temporal and spatial data. Our first task is to develop a model that reconstructs the full 2D field using only partial information of it (say coastal data only). The individual snapshots of sea level are treated here as images. The DL model operates in two stages. The initial stage comprises a convolutional autoencoder^31^, as illustrated in Fig. 2a. During the training phase, 2D images (sea level) are used as target data set. The encoder part of the model includes convolutional, pooling, flattening, and dense layers. In the decoder part, convolutional, up-sampling, dense, and reshape layers are utilized.

The convolutional method enables the extraction of specific spatial features from the data, including texture information^32^. Convolutional operations are a set of processes that extract features from images using convolutional filters, also known as kernels. The generation of feature maps is enabled by the application of kernels to the shifting of images. The kernel size is defined as a small matrix in the parameter selection process.

Pooling facilitates the reduction of spatial dimensionality, thereby reducing complexity^33^. The maxpooling layer was used in the pooling process, with a window size of (2,2). Maxpooling enables the acquisition of the maximum pixel value within the designated window during the execution of the sliding window operation.

The restoration of spatial resolution is achieved through up-sampling, a process that operates in a direction counter to that of pooling. The up-sampling factor in the up-sampling layers has been set to (2, 2), indicating that the width and length of the data entering the layer has been doubled.

Flatten is a function that converts a multidimensional input into one-dimensional vector. The Reshape layer is employed to modify the shape of the input, that is, to transform the input back into a 2-dimensional image. The dense layers refer to a standard fully connected neural network layer.

#### Forecasting

The second stage of the implemented model is an LSTM-based architecture for predicting the sea level at time $t+1$, as illustrated in Fig. 2b. Initial experiments showed a significant accumulation of errors when using the single-stage ConvLSTM-based model during recursive multi-step prediction. Therefore, we opted for the two-stage architecture (CNN-based autoencoder + LSTM), which produced more stable results.

Observations from the past (from time *t-n* to *t*) are used as an input for this study. To prepare the inputs of this architecture, latent spacings of the data were obtained by passing them through the encoder in the first stage. A total of 96 h (4 days) of past (hourly) observations are used. At this stage of the model’s development, its design incorporates LSTM and dense layers.

LSTM is a type of recurrent neural network (RNN) that was originally proposed by Hochreiter and Schmidhuber^34^. It is used to process time-dependent sequential data. LSTM networks have been identified as a suitable solution for time series forecasting. Their design enables the processing of sequential data while preserving relevant information over time, making them particularly well suited to predicting the next value in a series. The mechanism of the LSTM is characterized by using gates, which are structural components that manage the flow of information. These gates include input gates ${i}_{t}$, forget gates ${f}_{t}$, and output gates ${o}_{t}$, which determine what information is added to the cell state, what information is forgotten, and what information is output from the cell state, respectively. The following equations represent this mechanism:

5 $$i_{t} = \sigma \left( { w_{i} \left[ { H_{t - 1} , X_{t} } \right] + b_{i} } \right),$$

6 $$f_{t} = \sigma \left( { w_{f} \left[ { H_{t - 1} , X_{t} } \right] + b_{f} } \right),$$

7 $$o_{t} = \sigma \left( { w_{o} \left[ { H_{t - 1} , X_{t} } \right] + b_{o} } \right),$$

8 $$C_{t} = f_{t} \cdot C_{t - 1} + i_{t} \cdot \tanh \left( { w_{c} \left[ { H_{t - 1} , X_{t} } \right] + b_{c} } \right)$$

9 $$H_{t} = o_{t} \cdot\tanh \left( { C_{t} } \right),$$

The cell state ${C}_{t}$ can be considered a memory that stores information over time and controls the flow of information. ${C}_{t}$ and ${C}_{t-1}$ represent the cell state at the current and previous time steps, respectively. ${H}_{t}$ and ${H}_{t-1}$ represent the hidden states of the current and previous time steps, respectively. ${X}_{t}$ denotes the input at time *t*, ${w}_{*}$ is the weight matrix for a specific gate, ${b}_{*}$ is the corresponding bias for that same gate, and $\sigma$ the sigmoid function.

The reconstruction and prediction work are executed using the Python programming language and the TensorFlow library for DL operations. The selection of model parameters affects the performance and efficiency of the model. We employed the open-source framework Optuna, which was developed for the purpose of automatic hyperparameter optimization^35^, to identify the optimal parameters.

Optimization algorithms are employed to adapt the model weights and minimize the loss function (mean square error in this study). The learning rate of the optimization algorithm determines the step size of these updates. The optimizer employed here is Adam (Adaptive Moment Estimation)^36^. The activation function allows the network to identify complex patterns by applying nonlinear transformations to the input data^33^. The number of training samples used in a single iteration of the model learning process is referred to as the batch size^33^.

The initial 12 months of the 13-month dataset were allocated for training and validation purposes. Ten percent of the data set was used for validation, with the remaining portion allocated for training. The selected parameters of models are detailed in Table 2. Model training for the reconstruction experiment was also performed with the parameters in Table 2A.

**Table 2:** Hyperparameters used for experiments.

(A) **Stage 1 (AE)**		(B) **Stage 2 (Lstm-based)**	
**Hyperparameters**	**Value**	**Hyperparameters**	**Value**
Convolution filters	64,64,64,64,1, 64,64,64,64,1	Lstm Filters	32,64,480
Dense filters	256, 1536	Dense Filters	320, 256
Kernel size	(7,7)	Activation function (lstm)	Tanh
Activation function (dense)	LeakyReLU (alpha: 0.01), ReLu	Recurrent Activation function (lstm)	Sigmoid
Activation function (conv)	LeakyReLU (alpha: 0.01), linear (only the last layer)	Activation function (dense)	ReLU, linear (only the last layer)
Kernel initializer	Lecun normal, Glorot uniform (only the last layer)	Kernel initializer	He normal, Glorot uniform (only the last layer)
Learning rate	0.0005578	Learning Rate	0.002254
${\upbeta }_{1 },{\upbeta }_{2}$(Adam)	0.92196, 0.9740	${\upbeta }_{1 },{\upbeta }_{2}$(Adam)	0.95879, 0.98258
Batch size	16	Batch Size	64
Epoch	115	Time step	96
		Epoch	130
		Additional layer (dense) for FRT-A Filters Activation function Kernel initializer	128 tanh Glorot uniform

## Conclusions

This study demonstrates that DL methods can reconstruct and forecast basin-wide sea-level variability with skill comparable to traditional approaches, provided that suitable training data are available. The results reveal how prediction errors evolve with increasing spatial and temporal complexity of data and indicate the conditions under which DL models remain robust. By progressively increasing the complexity of the input signals, from monochromatic waves to full tidal datasets, we assessed the predictive skill and limitations of DL architecture, particularly CNNs and LSTM models.

Idealized experiments confirmed that DL models can achieve highly accurate short-term forecasts with minimal error accumulation, particularly for low-steepness waveforms. When applied to realistic tidal signals, the models maintained strong predictive skill over lead times up to 48 h, although forecast errors increased with higher spatial and temporal complexity of the signal.

Incorporating limited coastal observations improved predictions significantly, suggesting practical potential for hybrid systems combining real-time data and ML forecasting. The study also highlighted that DL models are sensitive to the steepness and spatial inhomogeneity of the sea-level signal, which affects their long-term forecasting capability.

An important finding is that DL models can reconstruct basin-wide sea level fields using only a limited number of coastal tide gauge observations. This capability highlights their potential for operational use, especially in data-rich coastal regions where rapid, computationally efficient forecasts are needed.

These results support the feasibility of using DL for operational sea-level forecasting in coastal environments and point toward its value in supplementing traditional numerical models, particularly where dense observation networks are available.

Future developments should aim at integrating real-time observational data (e.g., tide gauges, satellite measurements), extending the framework to storm surge and extreme event forecasting, and developing hybrid approaches that combine DL with physics-based models for improved reliability and interpretability.

## Supplementary Information

Below is the link to the electronic supplementary material.

Supplementary Material 1

Supplementary Material 2

Supplementary Material 3

Supplementary Material 4

Supplementary Material 5

Supplementary Material 6

Supplementary Material 7

Supplementary Material 8

Supplementary Material 9

Supplementary Material 10

## Data Availability

Data will be provided upon request.
