# Supplementary figures and images for: From monochromatic waves to realistic tides: deep learning for short-term forecasting of coastal ocean
Source: Sci Rep. 2025 Dec 21;15:44229. doi: 10.1038/s41598-025-31670-2 (PMC12722372; doi:10.1038/s41598-025-31670-2)

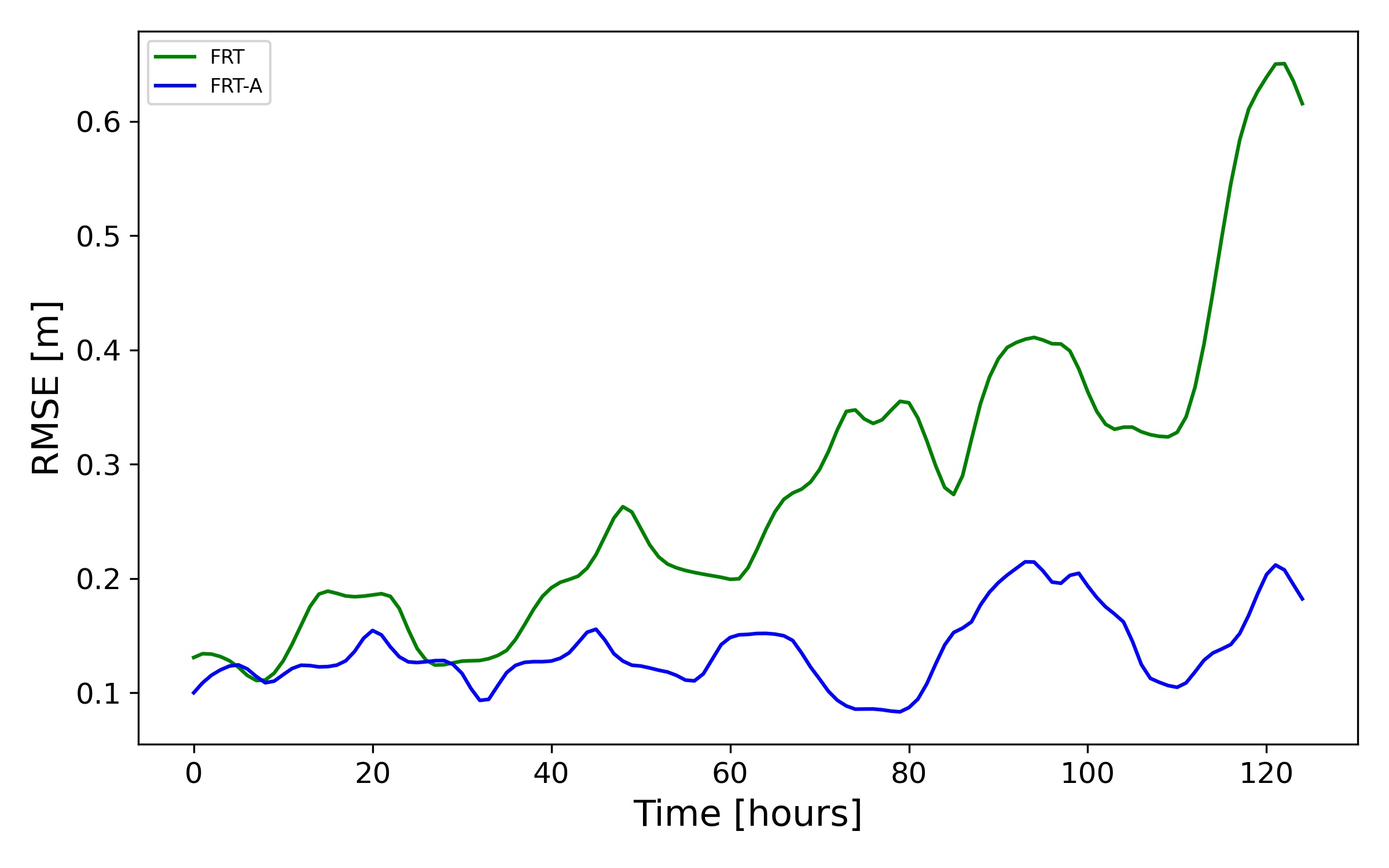

Supplement: Supplementary file 10 — Supplementary Material 10 [file 41598_2025_31670_MOESM10_ESM.jpg]
